# The Prevalence of Clinical Features in Patients with Aarskog–Scott Syndrome and Assessment of Genotype-Phenotype Correlation: A Systematic Review

**DOI:** 10.1155/2021/6652957

**Published:** 2021-02-02

**Authors:** Victor Zanetti Drumond, Lucas Sousa Salgado, Camila Sousa Salgado, Vitor Augusto de Lima Oliveira, Eliene Magda de Assis, Michel Campos Ribeiro, Analina Furtado Valadão, Alfredo Orrico

**Affiliations:** ^1^Dentistry School, Faculdade Pitágoras de Ipatinga, Ipatinga, Minas Gerais, Brazil; ^2^Medical School, União Educacional do Vale do Aço (UNIVAÇO), Ipatinga, Minas Gerais, Brazil; ^3^Department of Pediatrics and Neonatology, Hospital Unimed BH, Betim, Minas Gerais, Brazil; ^4^Faculdades Integradas do Norte de Minas-Ipatinga (FUNORTE), Ipatinga, Minas Gerais, Brazil; ^5^Department of Oral and Maxillofacial, Hospital Márcio Cunha, Ipatinga, Minas Gerais, Brazil; ^6^Interdipartimental Program for Molecular Diagnosis and Characterization of Pathogenic Mechanisms of Rare Genetic Diseases, Azienda Ospedaliero Universitaria Senese, Siena, Italy; ^7^Clinical Genetics, ASL Toscana Sudest, Ospedale della Misericordia, Grosseto, Italy

## Abstract

Aarskog–Scott syndrome is a genetically and clinically heterogeneous rare condition caused by a pathogenic variant in the FGD1 gene. A systematic review was carried out to analyse the prevalence of clinical manifestations found in patients, as well as to evaluate the genotype-phenotype correlation. The results obtained show that clinical findings of the craniofacial, orthopaedic, and genitourinary tract correspond to the highest scores of prevalence. The authors reclassified the primary, secondary, and additional criteria based on their prevalence. Furthermore, it was possible to observe, in accordance with previous reports, that the reported phenotypes do not present a direct relation to the underlying genotypes.

## 1. Introduction

Aarskog–Scott syndrome (AAS), also known as faciogenital dysplasia, is a rare X-linked syndrome with a recessive mode of inheritance (OMIM #305400). The condition was first described by Aarskog in 1970 and then detailed by Scott in two different families with multiple affected males [1, 2]. Aarskog related short-statured individuals with craniofacial anomalies such as hypertelorism, short nose, ptosis, and genital dysmorphism such as shawl scrotum and cryptorchidism. Scott, on the other hand, described the same characteristics in three different patients in 1971. In the following years, several other authors reported similar cases, describing patients whose phenotypes were characterised by the presence of variously associated signs, such as clinodactyly, brachydactyly, long philtrum, widow's peak, camptodactyly, interdigital webbing, and inguinal/umbilical hernia. These supported the identification of a nosologically distinct condition [3, 4]. In addition, various degrees of neurocognitive disabilities and/or behavior disorders were reported, ranging from attention deficit and hyperactivity disorder (ADHD) to severe intellectual disability. However, in most cases, the AAS-affected individuals showed average IQ [5–8]. In 1993, Teebi attempted to order the set of clinical signs documented in these patients and to outline a clinical filter for the diagnostic hypothesis. This study analysed and segmented the phenotypes of the reported cases into primary, secondary, and additional criteria, asserting that the presence of three or more classical signs could lead to a clinical suspicion of AAS [9].

The responsibility of FGD1 gene variants in the pathogenesis of the X-linked form of AAS (OMIM*∗*300546) was first described by German [10], based on translocation breakpoint analysis in a family. This gene maps to the short arm of the X chromosome (Xq11.22) and encodes a guanine nucleotide exchange factor (GEF). The GEF then activates Cdc42, participating directly in cytoskeletal organisation, growth regulation, and normal embryonic development in all mammals [11–13].

To date, 52 different pathogenic variants in FGD1 have been reported in AAS throughout all genes [14]. Although a correlation between the variants and the spectrum of clinical expression in AAS patients has been intensively investigated, no clear phenotype-genotype correlation has been characterised [15]. The syndrome is clinically and genetically heterogeneous; besides FGD1, other genes that have not yet been characterised could be involved in the pathogenesis. The low detection rate could be explained by cases that have been clinically diagnosed but with no supporting molecular data [8].

It is clear that knowing the most prevalent phenotypes is of paramount importance in facilitating the management of these patients. Therefore, we carried out a systematic review of published articles in order to summarise the manifestations in patients with positive genetic testing (X-linked “bona fide” AAS), appraising the quality of evidence available and analysing the possible genotype-phenotype correlation.

## 2. Materials and Methods

### 2.1. Search Methods and Eligibility Criteria

This systematic review was designed following the concepts of Preferred Reporting Items for Systematic Reviews and Meta-Analyses (PRISMA) [16]. A preplanned and comprehensive electronic bibliographic search was carried out, without date restrictions, in the main research databases, namely, Medline (via PubMed), SciELO, LILACS, and Latindex. The following search terms were used: “Faciogenital Dysplasia” OR “Faciodigitogenital syndrome” OR “Aarskog-Scott Syndrome” OR “Aarskog syndrome.”

The selected studies were those that met the following inclusion criteria: articles published in English; case reports that presented a complete description of the patients; male patients only; and cases that had a diagnosis confirmed by genetic testing, showing some types of pathogenic variants in the FGD1 gene. Incomplete articles, book chapters, cases without genetic proof, systematic reviews, meta-analyses, and any articles that did not meet all of the eligibility criteria were excluded.

### 2.2. Data Gathering and Statistical Analysis

Initially, a manual search was performed through the databases, and after applying the eligibility criteria, articles were selected for full reading. After checking each article, a list of those selected was created, separating them by title, author, and year of publication. The listing process was performed using Excel 2019 software (Microsoft Corporation, Redmond, Washington, USA).

Articles found through manual search were compared with the cases listed in the Human Gene Mutation Database (HGMDⓇ) [14]. Through the HGMDⓇ, the FGD1 gene was searched, with 52 pathogenic variants found. This finding was compatible with the results of the manual search using the keywords listed above as well as the eligibility criteria. Based on the classification developed by the HGMDⓇ, cases classified as DM? (denotes mutations reported as likely disease-causing, but with questionable pathogenicity) were excluded and only the cases marked as DM (indicates mutation reported to be disease-causing) were finally included.

After searching the databases and matching with the HGMDⓇ, the following information was taken from each study: phenotypic characteristics of each patient reported; age (when given); nationality (when given); and type of pathogenic variant and molecular constituents involved in the pathogenesis of variants, as shown in Table 1.

The findings were listed in a spreadsheet, showing the frequency of each phenotype by patient, as described by each author. The data were entered into two independent worksheets by two authors (L.S. and V.Z.). Once completed, they were cross-checked and analysed to search for differences, which if found were resolved by a third author (A.F.), who did a complete read-through of the relevant article. Finally, the database was meticulously checked again by the third author. The order of the listed phenotypes respects the criteria proposed in [9] and complemented in [3,15]. For each phenotype described by an author and present in the spreadsheet, a value was assigned, following Boolean logic: 1 for a finding and 0 for the absence of a finding. Using SPSS 23.0 software (IBM, Armonk, New York, USA), we obtained the absolute frequency of phenotypes per patient, the prevalence (percentage) in relation to the total number of cases, and the average/standard age deviation.

### 2.3. Bias and Quality Assessment

Case reports and case series are uncontrolled study designs known for an increased risk of bias. The quality assessment of the studies included in this work was performed using a validated tool for systematic reviews of case reports/case series proposed in [17,27–29]. As suggested by its authors, a general judgment of the quality of the methodology was preferable over an aggregate sum of the scores since more than one question was unsuitable for the papers included because these questions were related to adverse drug events. It is important to highlight that the quality assessment was not used as an exclusion criterion, due to the paucity of literature in this area. Even so, all of the articles included obtained a high and satisfactory evaluation for the purpose of this review, and they were all published in high impact factor journals with peer-review policies.

## 3. Results

**Figure 1:**
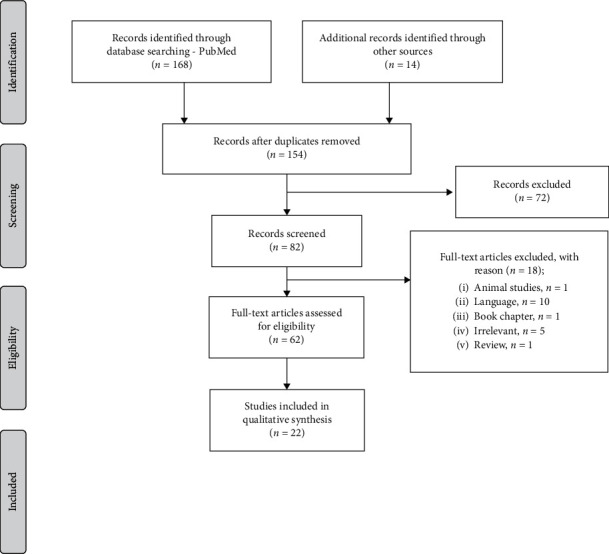
PRISMA flowchart showing the stages of the systematic review. The screening process identified 22 studies from an initial pool of 154 as being relevant to the current review and having satisfied the inclusion criteria.

One hundred and sixty-eight articles were found in the PubMed database and 14 in other search databases (LILACS and SciELO). In the search performed in the Latindex library, no article was listed. In total, 182 articles were collected and, after excluding duplicates, 154 remained. Another selection was performed considering the title and abstract, in which 72 were excluded, leaving 82. With the application of the eligibility criteria, a further 18 articles were excluded and 64 were selected for full reading (Figure 1). By the end of this process, 22 articles were included for analysis and qualitative synthesis, with 11 case reports/case series, three short reports, five mixed methods articles, and three research letters. It is noteworthy that these were the only levels of evidence available in the literature since it is a rare genetic syndrome.

The articles chosen for analysis (*n* = 22) were selected because they presented a complete phenotypic description as well as genetic testing, which confirmed the diagnosis of Aarskog–Scott syndrome. The sum of cases found in the articles was 58. From the 22 articles, 19 described ages, with a total of 43 patients, in which the average age was 10.6 (SD: 11.5 years old). Regarding the genotypes, 52 different pathogenic variants were described among the 58 cases, in which 33 patients presented with a point pathogenic variant (missense or nonsense mutations), representing about 56% of the total. In addition, 14 small deletions, four small insertions, three gross deletions, three splicing, and one duplication were described.

A total of 116 phenotypes were detected, which are grouped in Table 2 into primary (*n* = 11), secondary (*n* = 10), and additional (*n* = 9) categories based on the criteria proposed in [9] and complemented in [3,15]. Phenotypes not covered by this classification but described by other authors were allocated to the category “others” (*n* = 86). However, only the most prevalent are shown in Table 2. The compiled list of all phenotypes extracted can be found in the supplementary material, available on the Cambridge Core Online website.

### 3.1. Craniofacial Manifestations

**Figure 2:**
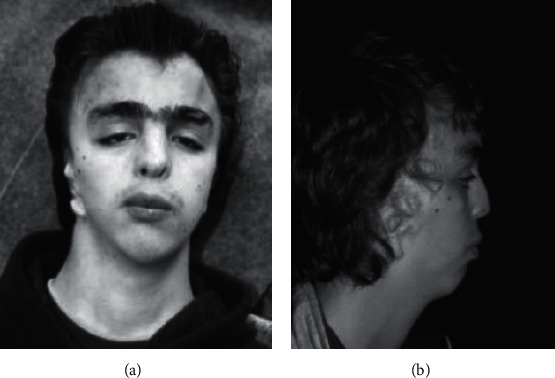
(a) Frontal view of a patient with severe expressions (image courtesy of Orrico et al. [38]). (b) Lateral view of a patient with severe expressions (image courtesy of Orrico et al. [38]).

Craniofacial manifestations were the most frequently described in the literature, representing 38.8% of all reported phenotypes, comprising 27.2% of the primary criteria, 40% of the secondary criteria, 44.4% of the additional criteria, and 39.5% of the others. Among the primary criteria, hypertelorism had a prevalence of 94.8% and was the most frequent phenotype among the 116. In addition, anteverted nostrils/short nose stand out with 75.9%, while bottom lip fold corresponded to only 5.2%. The main secondary phenotypes were ptosis (58.6%), widow's peak (46.6%), dysplastic ears (41.4%), and downward slanting palpebral fissures (39.7%). Among the additional criteria for craniofacial manifestations, long philtrum was the most prevalent (55.2%), followed by frontal bossing (27.6%), midface hypoplasia (20.7%), and lastly dental malocclusions (5.2%) (Figure 2).

The heterogeneity of the manifestations presented by patients with AAS is notorious, and uncommon phenotypes such as retrognathia (1.7%), preauricular tag (3.4%), and epicanthal fold (6.9%) are also described.

### 3.2. Orthopaedic Findings

Orthopaedic changes represented 18.1% of the 116 phenotypes, almost 50% less than craniofacial ones. Even with a lower frequency, orthopaedic findings represented 63.6% of the primary manifestations, with short stature the most prevalent in the group (82.8%), followed by brachydactyly/wide fingers (62.1%) and clinodactyly (43.1%). It is noteworthy that the “others” category has considerable variability; for example, it contains phenotypes such as metatarsus varus with a prevalence of 22.4%, while the prevalence of both coxa magna and arthrogryposis is just 1.7%.

### 3.3. Neurological and Ophthalmological Manifestations

The group with both neurological and ophthalmological manifestations comprised 25.8% of all phenotypic findings. Developmental delay was the most prevalent clinical finding, with 17.2%, followed by attention deficit and hyperactivity disorder at 10.3%. Apart from developmental delay, no other manifestation was listed as a primary, secondary, or additional criterion. The vast majority of the findings were listed in the other category and, despite having a relevant number of findings (*n* = 30), these were isolated with insignificant frequencies, for example, agenesis of corpus callosum, omphalocele, and sleep disorder, which in each finding had a percentage of 1.7%. This behavior corroborates with the premise of heterogeneity of the phenotypic findings of AAS.

Ophthalmological changes are not included by most authors as important criteria when suspected clinically. In this current analysis, in fact, they had a low prevalence, with astigmatism and strabismus being the most frequent, 5.2% of cases each, followed by myopia (3.4%) and amblyopia (1.7%).

### 3.4. Genitourinary Tract

Changes in the genitourinary system corresponded to only 8.6% of the total phenotypes. However, shawl scrotum, one of the primary criteria, had a prevalence of 79.3%, being the third most common criterion of all 116. Cryptorchidism is the only representative of this group among the secondary criteria, with a prevalence of 50%, while in the additional criteria, only hypospadias (1.7%) featured. In addition, reports of micropenis (3.4%), vesicoureteral reflux (1.7%), and hydrocele (1.7%) were recorded.

## 4. Discussion

Aarskog–Scott syndrome is an X-linked recessive genetic condition due to pathogenic variants in the FGD1 gene. AAS is also known as facio-digital-genital dysplasia [1–4]. The nomenclature is justified in view of the clinical findings since the intrinsic phenotypes of the craniofacial regions, upper limbs (hands), and genitourinary tract have considerably expressive prevalence rates, such as hypertelorism (94.8%), brachydactyly (62.1%), and shawl scrotum (79.3%). All three clinical findings are included within the primary criteria of the present study, as well as in the classification of criteria proposed in the literature [3,9,15]. Classically, the search for manifestations has been concentrated in these systems, even though a large number of neuropsychiatric findings are also clinically evaluated, with emphasis on attention deficit and hyperactivity disorder, which presented with a percentage of 10.3% in our study (Table 2).

To the best of our knowledge, etiopathogenesis is exclusively related to pathogenic variants that affect the FGD1 gene. However, the reported high frequency of failures in the detection of variants has been attributed to the heterogeneity of clinical findings, which leads to the possible involvement of other genes, even with different transmission modalities variously reported in the past (OMIM*∗*227330, 100050). Based on the analysis of phenotypes and pathogenic variants involved in all cases included in this study, overall it was not possible to define a clear genotype-phenotype correlation. Nevertheless, we consider it important to highlight that five members of the same family with a c.1341G>A; p.(Trp447*∗*) variant all presented with myopathic changes and distal arthropathy [25]. The importance of analysing phenotypic findings, especially those classified as primary, was addressed in [8], thus reinforcing the relevance and indispensability of formulating evidence-based criteria.

When it comes to AAS, differential diagnosis must be deeply investigated, considering that syndromes such as Noonan syndrome, SHORT syndrome, and Robinow syndrome have phenotypes very similar to those presented by AAS patients [30]. Manifestations such as hypertelorism, short stature, genital anomalies, and ptosis can overlap among these syndromes, thus reaffirming the paramount importance of knowing the most prevalent manifestations in patients with genetic testing confirmed to guide the suspected diagnosis [3,31]. In addition, clinical studies that indicate a high incidence of AAS should be considered cautiously since there is a strong inclusion bias, in which patients without a definitive diagnosis of AAS may have been mistakenly included.

Regarding affected females, they are often asymptomatic or may show milder forms of the syndrome, presenting phenotypes such as hypertelorism, widow's peak, and brachydactyly [20]. This fact was addressed in [10, 32–35], which related this behavior to the X-linked recessive disorder. Furthermore, the authors in [8, 20] point out the possibility of a pattern of X-chromosome inactivation. Female carriers present few phenotypes considered relevant when AAS is suspected, and therefore, these cases should be carefully investigated, both clinically and molecularly. The understanding of the relation between clinical and molecular manifestations in females will be extremely important in order to increase the detection rate of female cases in the future [8]. As for all X-linked recessive conditions, pathogenic variants in the FGD1 in female carriers are generally documented after the characterisation of the variant in a male relative. Thus, the few phenotypical signs presented by females are reported after, undoubtedly with an ascertainment bias.

One of the objectives of evidence-based practice is to provide the best management for patients. When surveying the articles available in the literature, the need to standardise the hierarchy of clinical criteria for Aarskog–Scott syndrome became clear. Although important, it is necessary to emphasise that this study is not proposing that the diagnosis of AAS be made exclusively clinical, as this has already been exhaustively addressed in the literature as ineffective, given the overlap of very similar phenotypes with other syndromes [36]. Overall, in this field as in others, a genome first approach is becoming prevalent, while FGD1 sequencing studies in suspected AAS cases are giving way to NGS, using panels of genes or exome or whole-genome approaches for the identification of molecular defects. Nevertheless, in our opinion, this should not prevent efforts to better identify the clinical manifestations shown by patients to be proposed for molecular studies. In our work, as a way of summarising the recorded clinical findings, the phenotypes were classified based on the percentages of prevalence found, so those in which the cutoff value was ≥50% were included among the primary criteria, between 30% and 49% as secondary criteria, and between 15% and 29% as additional criteria (Table 3). In this way, criteria such as long philtrum, previously described as additional, were reclassified as primary. Formerly secondary criteria such as ptosis, cryptorchidism, and joint hypermobility were raised to the primary category. In the same way that some phenotypes have moved up in the hierarchy, others have been downgraded, such as short/broad hands, interdigital webbing, and camptodactyly, which moved from primary to secondary criteria. Attention is drawn to the exclusion from the three categories of hypospadias, observed in only one case (1.7%) and bottom lip fold, described in just three cases (5.2%). In addition, some new criteria are suggested, in which syndactyly would cease to be primary, umbilical hernia would cease to be secondary, and both would be included in the additional category. Furthermore, some manifestations previously not included in any category would become additional, such as the case of metatarsus varus, crease below the lower lip, low set ears, round face, interphalangeal joint contracture, and ogival palate.

The organisation of clinical criteria based on the prevalence of phenotypic findings has the main objective of assisting the clinical suspicion of Aarskog–Scott syndrome. Summarising these findings is truly useful, especially for deficient health systems, such as those in underdeveloped countries, where access to genetic testing is difficult, whether for infrastructure or economic reasons, a scenario discussed by the WHO in 2006 [37].

Ascertainment bias often refers to situations in which the way data are collected is more likely to include some members of a population than others. This scenario happens when there is more intense surveillance or screening for the outcome of interest in certain populations. For example, phenotypes considered as primary criteria, such as hypertelorism, shawl scrotum, and short stature, may be overrepresented in our sample, as patients who present those phenotypes have a higher probability of being tested for pathogenic variants in the FGD1 gene. However, to the best of our knowledge, there are currently no studies reporting pathogenic variants in the FGD1 gene in cases analysed, for example, by whole-genome sequencing (WGS) or whole-exome sequencing (WES), and, therefore, retrospectively reported as AAS cases. Possibly, in the future, with the growth of data drawn through these approaches, we will have diagnoses of AAS with a poor phenotypic expression.

## 5. Conclusion

In conclusion, it was not possible to observe any evidence of a genotype-phenotype causal relationship between the type and position of the pathogenic variants with clinical expression, as previously observed by other authors. Furthermore, the organisation of clinical findings segregated according to their prevalence becomes truly useful for medical practice and management of patients with rare genetic diseases possibly to be directed to genetic tests. The need for a multidisciplinary approach from diagnosis to therapeutic interventions is also emphasised since the clinical manifestations affect diverse systems. Based on the exposed premises, the elaboration of more studies that seek to analyse the nature of clinical findings in light of a better understanding of the molecular mechanisms involved is suggested.

## Figures and Tables

**Table 1:** General information collected from case reports with genetic testing done.

Author	Year	Country	Patient	Age (years)	Type of pathogenic variant
Schwartz et al. [6]	2000	USA	1 (FC)	7	Missense/nonsense (1565G>A)
			2 (IT)	12	Missense/nonsense (1565G>A)
			3 (MK)	11	Gross deletion (Incl.ex.9-12)

Orrico et al. [17]	2010	Italy	4 (II1)	66	Missense/nonsense (1829G>A)
			5 (IV9)	9	Missense/nonsense (1829G>A)
			6 (IV10)	19	Missense/nonsense (1829G>A)

Orrico et al. [18]	2004	NR	7 (50)	29	Small insertion (528insC)
		NR	8 (90)	9	Small insertion (528insC)
		Portugal	9 (25)	11	Missense/nonsense (614G>T)
		NR	10 (53)	1.3	Small deletion (982delC)
		NR	11 (61)	21	Gross deletions (944-975del32)
		NR	12 (26)	3	Missense/nonsense (1193A>C)
		NR	13 (73)	4.5	Missense/nonsense (1328G>A)
		NR	14 (58)	1.6	Small deletion (1316-1319del AGCT)
		Ireland	15 (65)	16	Small deletion (2530delG)
Orrico et al. [18]	2004	Italy	16	16	Missense/nonsense (c.1223G>A)

Shalev et al. [19]	2012	Israel	17 (IV2)	24	Small deletion (c.2192delA)
			18 (IV3)	16	Small deletion (c.2192delA)
			19 (IV4)	2	Small deletion (c.2192delA)

Kaname et al. [11]	2006	Japan	20 (1)	13	Missense/nonsense (c.1327G>T)
			21 (2)	4	Missense/nonsense (c.2221G>T)
Bottani et al. [20]	2007	Switzerland	22	8	Missense/nonsense (c.1396A>G)

Orrico et al. [17]	2010	Italy	23	15	Small insertions (c.944dupC)
			24	6	Small insertions (c.944dupC)
Bedoyan et al. [21]	2009	USA	25	1.25	Gross deletions (entire gene)

Orrico et al. [18]	2004	NR	26 (9)	NR	Small deletions (806delC)
		NR	27 (2)	NR	Missense/nonsense (c.1205G>A)
		NR	28 (5)	NR	Missense/nonsense (c.1590T>A)
		NR	29 (10)	NR	Small deletions (1620delC)
		NR	30 (3)	NR	Missense/nonsense (c.1673C>G)
		NR	31 (11)	NR	Splicing (c.1935+3A>C)
		NR	32 (8)	NR	Missense/nonsense (c.1966C>T)
		NR	33 (6)	NR	Missense/nonsense (c.1966C>T)
		NR	34 (7)	NR	Missense/nonsense (c.1966C>T)
		NR	35 (1)	NR	Small deletions (2020_2022 del GAG)
		NR	36 (4)	NR	Missense/nonsense (c.2242A>G)

Pillozzi-Edmonds et al. [22]	2011	Canada	37 (1)	0.75	Missense/nonsense (c.175C>T)
			38 (2)	0.75	Missense/nonsense (c.175C>T)
Ronce et al. [2]	2012	France	39	NR	Duplication

Aten et al. [23]	2013	Netherlands	40 (III-1)	NR	Small deletions (c.2016-35delA)
			41 (III-2)	NR	Small deletions (c.2016-35delA)
			42 (I-1)	NR	Small deletions (c.2016-35delA)
Altincik et al. [24]	2013	Turkey	43	7	Splicing (c.482-2A>G)

Al-Semari et al. [25]	2013	Saudi Arabia	45 (index)	16	Missense/nonsense (c.1341G>A)
			46 (bro‡ 1)	14	Missense/nonsense (c.1341G>A)
			47 (bro 2)	21	Missense/nonsense (c.1341G>A)
			48 (nep 1)	1	Missense/nonsense (c.1341G>A)
			49 (nep 2)	4	Missense/nonsense (c.1341G>A)
Völter et al. [26]	2014	Germany	44	9	Missense/nonsense (c.1468C>T)
Niida et al. [4]	2014	Japan	50	6	Missense/nonsense (c.1340G>A)

Pérez-Coria et al. [3]	2015	Mexico	51 (1)	6	Missense/nonsense (c.1138G>T)
			52 (4)	12	Missense/nonsense (c.1990C>T)
			53 (5)	4	Missense/nonsense (c.1990C>T)
Ge et al. [13]	2015	China	54	2	Missense/nonsense (c.1270A>G)
Parıltay et al. [14]	2016	Turkey	55	14	Splicing mutations (c.1340+2T>A)
Griffin et al. [27]	2016	USA	56 (IV-1)	1	Missense/nonsense (c.2761C>T)

Hamzeh et al. [9]	2017	UAE	57 (IV-3)	7	Small deletions (c.53delC)
			58 (IV-5)	3	Small deletions (c.53delC)

NR: not reported; UAE: United Arab Emirates; bro: brother; nep: nephew. Note: in the “Patient” column, numbers outside the parentheses refer to how the present work classified the patients and numbers inside the parentheses correspond to how the author of the article referred to them.

**Table 2:** Prevalence of clinical features segregated into categories according to the literature.

Clinical features	*n* (%)
*Primary criteria*	58 (100)
Hypertelorism	55 (94.8)
Short stature	48 (82.8)
Shawl scrotum	46 (79.3)
Anteverted nostrils/short nose	44 (75.9)
Brachydactyly/wide fingers	36 (62.1)
Clinodactyly	25 (43.1)
Short/broad hands	23 (39.7)
Interdigital webbing	20 (34.5)
Camptodactyly	19 (32.8)
Syndactyly	14 (24.1)
Bottom lip fold	3 (5.2)

*Secondary criteria*	
Ptosis	34 (58.6)
Cryptorchidism	29 (50.0)
Joint hypermobility	29 (50.0)
Widow's peak	27 (46.6)
Dysplastic ears	24 (41.4)
Downward slanting palpebral fissures	23 (39.7)
Inguinal hernia	21 (36.2)
Wide feet	18 (31.0)
Umbilical hernia	9 (15.5)
Prominent umbilicus	4 (6.9)

*Additional criteria*	
Long philtrum	32 (55.2)
Simian creases	20 (34.5)
Frontal bossing	16 (27.6)
Midface hypoplasia	12 (20.7)
Developmental delay	10 (17.2)
Bone age retarded	7 (12.1)
Obesity	6 (10.3)
Dental malocclusion	3 (5.2)
Hypospadias	1 (1.7)

*Others*	
Metatarsus varus	13 (22.4)
Crease below the lower lip	11 (19.0)
Low set ears	11 (19.0)
Round face	10 (17.2)
Interphalangeal joint contracture	9 (15.5)
Ogival palatus	9 (15.5)
Pectus excavatum	8 (13.8)
Broad nasal ridge	7 (12.1)
Micrognathia	7 (12.1)
Short neck	7 (12.1)
ADHD	6 (10.3)

**Table 3:** Summarization of clinical features reclassified according to their prevalence.

Clinical features	*n* (%)
*Primary criteria (≥50%)*	58 (100)
Hypertelorism	55 (94.8)
Short stature	48 (82.8)
Shawl scrotum	46 (79.3)
Anteverted nostrils/short nose	44 (75.9)
Brachydactyly/wide fingers	36 (62.1)
Ptosis	34 (58.6)
Long philtrum	32 (55.2)
Cryptorchidism	29 (50.0)
Joint hypermobility	29 (50.0)

*Secondary criteria (30–49%)*	
Widow's peak	27 (46.6)
Clinodactyly	25 (43.1)
Dysplastic ears	24 (41.4)
Short/broad hands	23 (39.7)
Downward slanting palpebral fissures	23 (39.7)
Inguinal hernia	21 (36.2)
Interdigital webbing	20 (34.5)
Simian creases	20 (34.5)
Camptodactyly	19 (32.8)
Wide feet	18 (31.0)

*Additional criteria (15–29%)*	
Frontal bossing	16 (27.6)
Syndactyly	14 (24.1)
Metatarsus varus	13 (22.4)
Midface hypoplasia	12 (20.7)
Crease below the lower lip	11 (19.0)
Low set ears	11 (19.0)
Developmental delay	10 (17.2)
Round face	10 (17.2)
Umbilical hernia	9 (15.5)
Interphalangeal joint contracture	9 (15.5)
Ogival palatus	9 (15.5)

## Data Availability

The data used to support the findings of this study are available in the Supplementary Materials and can also be obtained from the corresponding author on request. Victor Zanetti Drummond and Lucas Sousa Salgado are joint first authors of the manuscript.

## Abbreviations

AAS: Aarskog–Scott syndrome
HGMDⓇ: The Human Gene Mutation Database
DM?: Mutations reported as likely disease-causing, but with questionable pathogenicity
DM: Mutation reported to be disease-causing
SD: Standard deviation
WGS: Whole-genome sequencing
WES: Whole-exome sequencing.

## Supplementary Materials

Supplementary Materials The prevalence of clinical features in patients with Aarskog–Scott syndrome.
