# Supplementary material for: The Prevalence of Clinical Features in Patients with Aarskog–Scott Syndrome and Assessment of Genotype-Phenotype Correlation: A Systematic Review
Source: Genet Res (Camb). 2021 Feb 2;2021:6652957. doi: 10.1155/2021/6652957 (PMC7953535; doi:10.1155/2021/6652957)
Supplement: Supplementary Materials — The prevalence of clinical features in patients with Aarskog–Scott syndrome. [file 6652957.f1.pdf]

Genetics Research. The prevalence of clinical features in patients with Aarsk

Victor Zanetti Drumond, Lucas Sousa Salgado, Camila Sousa Salgado, Vitor Augu

Supplementary Table S1: L

Authors	Year	Country	Patient	Age(years)	Phenotypes
**Schwartz et al. [3]**	2000	USA§	1(FC)	7	Brachydactyly/wide fingers; Cupid's Bow; Dorsal Groove penis; Downward Slant Palpebral Fissures; Hypertelorism ; Interphalangeal Joint Contracture; Joint Hypermobility; Long Philtrum; Macrocephaly; Pointed Chin.; Ptosis; Short Stature; Simian Creases; Umbilical Hernia
			2(IT)	12	Brachydactyly/wide fingers; Cupid's Bow; Downward Slanted Palpebral Fissures; Hypertelorism; InguiNA†l Hernia; Interphalangeal Joint Contracture; Joint hypermobility; Long Philtrum; Macrocephaly; Pointed Chin; Ptosis; Shawl Scrotum; Short Stature; Simian Crease
			3(MK)	11	Anteverted Nostrils; Brachydactyly/Wide Fingers; Hypertelorism; Shawl Scrotum; Short Stature; Syndactyly; Cryptorchidism; Downward slant palpebral fissures; InguiNA†l hernia; Ptosis; Widow's peak; Bone Age retarded; Myopia

**Orrico et al. [37]**	2000	Italy	4(II1)	66	Anteverted Nostrils; Camptodactyly; Clinodactyly; Crase Below Lower Lip; Cryptorchidism; Downward Slanted Palpebral Fissures; Hypertelorism; InguiNA†l Hernia; Interdigital Webbing; Ptosis; Shawl Scrotum; Short Stature; Short/Broad Hands; Umbilical Hernia; Widow's Peak
			5(IV9)	9	Anteverted Nostrils; Anteverted nostrils/Short Nose; Clinodactyly; Crease Below Lower Lip; Cryptorchidism; Downward Slant Palpebral Fissures; Dysplastic Ears; Frontal Bossing; Hypertelorism; InguiNA†l Hernia; Interdigital Webbing; Joint Hypermobility; Long Philtrum; Midface Hypoplasia; Ptosis; Round Face; Shawl Scrotum; Short Stature; Short/Broad Hands; Umbilical Hernia; Wide feet; Widow's peak
			6(IV10)	19	Anteverted Nostrils; Anteverted nostrils/Short Nose; Clinodactyly; Crease Below Lower Lip; Cryptorchidism; Downward Slant Palpebral Fissures; Hypertelorism; InguiNA†l Hernia; Interdigital Webbing; Joint Hypermobility; Ptosis; Shawl Scrotum; Short Stature; Short/Broad Hands; Umbilical Hernia; Widow's Peak

		NR*	7(50)	29	Anteverted Nostrils; Anteverted nostrils/Short Nose; Brachydactyly/Wide Fingers; Camptodactyly; Clinodactyly; Cryptorchidism; Hydrocele; Hypertelorism; InguiNA†l Hernia; Joint Hypermobility; Long Philtrum; Oligoasthenozoospermia; Ptosis; Shawl Scrotum; Short Stature; Syndactyly
		NR*	8(90)	9	Anteverted Nostrils; Brachydactyly/Wide Fingers; Camptodactyly; Cryptorchidism; Downward Slant Palpebral Fissures; Dysplastic Ears; Frontal Bossing; Hypertelorism; InguiNA†l Hernia; Interdigital Webbing; Joint Hypermobility; Long Philtrum; Ptosis; Round Face; Shawl Scrotum; Short Stature; Short/Broad Hands; Syndactyly; Widow's Peak
		Portugal	9(25)	11	Anteverted Nostrils; Brachydactyly/Wide Fingers; Camptodactyly; Cryptorchidism; Downward Slant Palpebral Fissures; Dysplastic Ears; Frontal Bossing; Hypertelorism; InguiNA†l Hernia; Interdigital Webbing; Joint Hypermobility; Long Philtrum; Ptosis; Round Face; Shawl Scrotum; Short Stature; Short/Broad Hands; Syndactyly; Widow's Peak

**Orrico et al. [6]**	2004	NR*	10(53)	1.3	Anteverted nostrils/Short Nose; Brachydactyly/wide fingers; Clinodactyly; Cryptorchidism; Downward slant palpebral fissures; Hypertelorism; InguiNA†l hernia ; Joint hypermobility; Long Philtrum;Shawl Scrotum ;Short stature; Simian Crease; Syndactyly; Umbilical hernia; Widow’s peak
		NR*	11(61)	21	Behavioral Disorders;Brachydactyly/wide fingers; Camptodactyly; Hypertelorism; Joint hypermobility;Ptosis; Shawl Scrotum; short neck; Short stature; Social/Language skills delayed; Strabismus; Syndactyly
		NR*	12(26)	3	Anteverted nostrils/Short Nose;Anteverted nostrils/Short Nose; Brachydactyly/wide fingers; Clinodactyly; Epicanthal fold; Hypertelorism ; InguiNA†l hernia; Joint hypermobility; Long philtrum; Metatarsus varus; Round face; Shawl Scrotum; Short stature;Syndactyly; Umbilical hernia; Widow’s peak
		NR*	13(73)	4.5	Anteverted nostrils/Short Nose; Bone age retarded; Camptodactyly; Cleft Lip Palate; Downward slant palpebral fissures; Epicanthal fold; Hypertelorism; InguiNA†l Hernia; Joint Hypermobility; Long Philtrum; Low set ears; Ptosis; Shawl Scrotum; Syndactyly; Widow's peak

		NR*	14(58)	1.6	ADHD; Anteverted nostrils/Short Nose; Axial Hypotonia;Brachydactyly/Wide fingers; Cryptorchidism; Development delayed; Hypertelorism; Joint Hypermobility; Long Philtrum; Low set ears; Prominent umbilicus; Ptosis; Shawl Scrotum; Short Stature; Syndactyly
		Ireland	15(65)	16	Anteverted nostrils/Short Nose;Brachydactyly/Wide Fingers; Camptodactyly; Cryptorchidism; Downward slant palpebral fissures; Hypertelorism; Long philtrum; Ptosis; Shawl Scrotum; Short/Broad Hands; Syndactyly; Wide feet
**Orrico et al. [7]**	2005	Italy	16	16	ADHD; Axial hypotonia; Behavioral disorder; Borderline mental retardation;Brachydactyly/Wide fingers; Broad NA†sal bridge; Downward slant palpebral fissures; Dysplastic ears; Enlarged Virchow-Robin spaces; HyperNA†sal speech; Hypertelorism; Interdigital webbing; Joint hypermobility; Low set ears; MicrogNA†thia; Midface Hypoplasia; Ogival palate; Pectus excavatum; Prominent umbilicus; ptosis; Short stature; Simian Creases; Strabismus

**Shalev et al. [24]**	2006	Israel	17(IV2)	24	Anteverted nostrils/Short Nose; Brachydactyly/Wide fingers; Camptodactyly; Dysplastic ears; Hypertelorism; Long philtrum; Pectus cariNA†tum; Ptosis; Round face; Shawl Scrotum Short stature; Simian creases; Syndactyly
			18(IV3)	16	Brachydactyly/wide finger; Camptodactyly; Dysplastic ears; Frontal bossing; Hypertelorism; Ptosis; Round face; Short Stature; Simian creases; Syndactyly
			19(IV4)	2	Anteverted nostrils/Short Nose; Behavioral disorder; Development delayed; Fine and gross motor skills delayed; Hypertelorism; Microcephaly, Neurocognitive development delayed; Ptosis; Seizures; Short stature; Social/Language Skills delayed; Vertigo
**KaNA†me et al. [8]**	2006	Japan	20(1)	13	ADHD;Brachydactyly/Wide fingers; Frontal bossing; Hypertelorism; Interdigital webbing; Interphalangeal joint contracture; Joint hypermobility; Ptosis; Shawl scrotum; Short stature; Short/Broad Hands; Wide feet
			21(2)	4	Brachydactyly/Wide fingers; Frontal Bossing; Hypertelorism; Interdigital Webbing; Interphalangeal joint contracture; Joint hypermobility; Shawl Scrotum; Short stature; Short/Broad Hands; wide feet

**Bottani et al. [25]**	2007	Switzerland	22	8	Clinodactyly; Clubbed feet;Cryptorchidism; InguiNA†l hernia; Joint hypermobility; Pectus excavatum; Seizures; Shawl Scrotum; Unilateral focal frontoparietal PMG, Migraine headaches
**Orrico et al. [26]**	2007	Italy	23	15	Agenesis of Corpus Callosum; Anteverted nostrils/Short Nose;Brachydactyly/wide fingers; Crease below lower lip; Downward slant palpebral fissures ; Dysplastic ears; Fine and gross motor skills delayed; Hydrocele; Hypertelorism ; InguiNA†l hernia ; Interdigital webbing; Long philtrum; Midface Hypoplasia; Neurocognitive development delayed; Omphalocele; Parietal-occipital encephalocele; Pectus excavatum; Ptosis; Right hemifacial microsomia; Shawl Scrotum; Short neck; Short stature; Short/Broad Hands; Simian Creases; still's murmur ; Submucous cleft palate; Synophry; Transethmoidal;Meningoencephalocele; Ventricular septal defect; Wide feet;Widow's Peak
			24	6	Anteverted nostrils/Short Nose; Bifid uvula; Brachydactyly/Wide fingers; Camptodactyly; Crease below lower lip; InguiNA†l hernia; Interdigital webbing; Long philtrum; Low set ears; Metatarsus varus; Pectus excavatum; Prominent umbilicus; Ptosis; Round face; Shawl Scrotum; Short neck; Short stature; Simian Creases; Synophrys; Telecanthus; Widow's peak

**Bedoyan et al. [27]**	2009	USA§	25	1.25	Anteverted nostrils/Short Nose; Astigmatism; Camptodactyly; Cryptorchidism; Downward slant palpebral fissures; Dysplastic ears; Hypertelorism; InguiNA†l hernia; Ogival palate; Ptosis; Shawl Scrotum
		NR*	26(9)	NR*	Anteverted nostrils; Brachydactyly/Wide fingers; Clinodactyly; Cryptorchidism; Dental malocclusion; Dysplastic ears; Hypertelorism; long philtrum; Midface hypoplasia; Obesity; Shawl scrotum; Short stature; Short/Broad Hands; Wide feet
		NR*	27(2)	NR*	Anteverted nostrils/Short Nose; Bone age retarded; Development delayed; Dysplastic ears; Hypertelorism; Obesity; preauricular tag; Shawl scrotum; Short stature; Short/Short/Broad Hands; Simian creases; Widow's peak
		NR*	28(5)	NR*	Anteverted nostrils/Short Nose;Camptodactyly; Cryptorchidism; Development delayed; Hypertelorism; InguiNA†l hernia; Interdigital webbing; Long philtrum; Metatarsus varus; Ptosis; Shawl scrotum; Short stature; Short/Broad Hands; Simian creases; Umbilical hernia; Wide feet; Widow's peak

Orrico et al. [16]	2010	NR*	29(10)	NR*	Anteverted nostrils/Short Nose; Brachydactyly/wide fingers; Camptodactyly; Clinodactyly; Crease below lower lip; Downward slant palpebral fissures; Fetal Nuchal Oedema; Frontal bossing; Hypertelorism; Interdigital webbing; Long philtrum; Metatarsus varus; Microcephaly; Ptosis; Short stature; Short/Broad Hands; Simian creases; Wide feet
		NR*	30(3)	NR*	Anteverted nostrils/Short Nose; Brachycephaly; Brachydactyly/wide fingers; Clinodactyly; Crease below lower lip; Development delayed; Dysplastic ears; Hypertelorism; Hypospadias; InguiNA†l hernia; Long Philtrum; Micropenis; Midface hypoplasia; Obesity; Ptosis; Shawl scrotum, Short stature; Short/Short/Broad Hands; Umbilical hernia; Widow's peak
		NR*	31(11)	NR*	Anteverted nostrils/Short Nose; bone age retarded; Brachydactyly/Wide fingers; Clinodactyly; crease below lower lip; downward slant palpebral fissures; frontal bossing; Hypertelorism; long philtrum; midface hypoplasia; Patent ductus arteriosus; prominent umbilicus; ptosis; shawl scrotum; Short Stature;Short/Broad Hands; wide feet; widow's peak
		NR*	32(8)	NR*	Anteverted nostrils/Short Nose;Anteverted nostrils/Short Nose; Brachydactyly/wide fingers;Camptodactyly; Clinodactyly; Cryptorchidism; Hypertelorism; long philtrum; obesity; Shawl scrotum; Short stature; Short/Broad Hands; Wide feet

		NR*	33(6)	NR*	Anteverted nostrils/Short Nose; bone age retarded; Brachydactyly/Wide fingers; Camptodactyly; clinodactyly; crease below lower lip; cryptorchidism; downward slant palpebral fissures; dysplastic ears; hypertelorism; joint hypermobility; shawl scrotum; Short stature; Short/Broad Hands; simian creases; Strabismus; wide feet; widow's peak
		NR*	34(7)	NR*	Anteverted nostrils/Short Nose; Brachydactyly/wide fingers; Clinodactyly; Congenital glaucoma; crease below lower lip; cryptorchidism; Dysplastic ears; frontal bossing; hypertelorism; Interdigital webbing; Joint hypermobility; long philtrum; metatarsus varus; midface hypoplasia; Shawl scrotum; short stature;Short/Broad Hands; Wide feet; Widow's peak
		NR*	35(1)	NR*	Anteverted nostrils/Short Nose;Anteverted nostrils/Short Nose; bone age retarded; crease below lower lip; downward slant palpebral fissures; dysplastic ears; Exostosis; hypertelorism; large eyebrows; long philtrum; pectus excavatum; shawl scrotum; short stature; Short/Broad Hands; wide feet; widow's peak
		NR*	36(4)	NR*	Anteverted nostrils/Short Nose;Brachydactyly/wide fingers; camptodactyly; clinodactyly; clubbed feet; Cryptorchidism; development delayed; Frontal bossing; hypertelorism; interdigital webbing; Shawl Scrotum; Short stature; Short/Broad Hands; Widow's peak

**Pillozzi-Edmonds et al. [28]**	2011	CaNA†da	37(1)	0.75	Axial hypotonia; bitemporal NA†rrowing;Clinodactyly; clubbed feet; frontal bossing; hydronephrosis; hypertelorism; inguiNA†l hernia; interphalangeal joint contractures; low set ears; preauricular tag; Shawl scrotum; simian creases; vesicoureteral reflux
			38(2)	0.75	Anteverted nostrils/Short Nose; Axial Hypotonia; broad NA†sal bridge; clinodactyly; clubbed feet; cryptorchidism; frontal bossing; hypertelorism; interphalangeal joint contracture; long philtrum; low set ears; myopia; preauricular tag; Shawl Scrotum. InguiNA†l Hernia; Simian Creases; thin upper lip vermillion; triangular downturned mouth; upslanted palpebral fissures; vesicoureteral reflux
**Ronce et al. [29]**	2012	France	39	NR*	Anteverted nostrils/Short Nose; arthrogryposis;Camptodactyly; cryptorchidism; dysplastic ears; hypertelorism; joint hypermobility; large fontanelle/sutures; long philtrum; low set ears; metatarsus varus; micropenis; midface hypoplasia; RetrogNA†thia; round face; short neck; Short/Broad Hands; simian creases; wide feet; widow's peak
			40(III-1)	NR*	Anteverted nostrils/Short Nose; cleft lip palate; cow's lick; cryptorchidism; dysplastic ears;Hypertelorism; interphalangeal joint contracture; joint hypermobility; long philtrum; shawl scrotum; short stature; Short/Broad Hands; wide feet

					[illegible]
**Aten et al. [30]**	2012	Netherlands	41 (III-2)	NR*	Anteverted nostrils/Short Nose; cow's lick; cryptorchidism; dysplastic ears;Hypertelorism; joint hypermobility; long philtrum; shawl scrotum; short stature; Short/Broad Hands; widow's peak
			42 (I-1)	NR*	Cow's lick;Hypertelorism; Interphalangeal joint contractures; long philtrum; Short Stature; Widow's peak
**Altıncık et al. [31]**	2013	Turkey	43	7	Amblyopia; astigmatism; borderline mental retardation;Brachydactyly/wide fingers; broad NA†sal bridge; cryptorchidism; Frontal bossing; Hypertelorism; interdigital webbing; long philtrum; short stature; Short/Broad Hands
**Völter et al. [32]**	2014	Germany	44	9	ADHD; Broad NA†sal bridge; cleft lip palate;Clinodactyly; hemianopsia; hyperNA†sal speech; hypertelorism; interdigital webbing; long philtrum; low set ears; metatarsus varus; microcephaly; Midface hypoplasia; NA†sal ectopic teeth; ptosis; round face; short neck; Short stature; Short/Broad Hands; telecanthus; Von Willebrand disease
			45(Index)	16	Anteverted nostrils/Short Nose; Anteverted nostrils/Short Nose; hypertelorism; joint hypermobility; metatarsus varus; MicrogNA†thia; Myopathy; ogival palate; ptosis; shawl scrotum; short stature

**Al-Semari et al. [19]**	2013	Saudi Arabia	46(Bro1‖)	14	Anteverted nostrils/Short Nose; Anteverted nostrils/Short Nose; hypertelorism; joint hypermobility; metatarsus varus; microgNA†thia; ogival palate; pectus excavatum; shawl scrotum; short stature
			47(Bro2‖)	21	Anteverted nostrils/Short Nose; Anteverted nostrils/Short Nose; hypertelorism; joint hypermobility; metatarsus varus; microgNA†thia; ogival palate; shawl scrotum; short stature
			48(Nep¶1)	1	Anteverted nostrils/Short Nose; Anteverted nostrils/Short Nose; hypertelorism; joint hypermobility; metatarsus varus; MicrogNA†thia; ogival palate; pectus excavatum; ptosis; shawl scrotum; short stature
			49(Nep¶2)	4	Anteverted nostrils/Short Nose; Anteverted nostrils/Short Nose; coxa magNA†; hypertelorism; joint hypermobility; metatarsus varus; microgNA†thia; myopathy; ogival palate; shawl scrotum; short stature
**Niida et al.[33]**	2014	Japan	50	6	Anteverted nostrils/Short Nose; brachydactyly/wide fingers; clinodactyly; cryptorchidism; hypertelorism; interdigital webbing; ptosis; round face; shawl scrotum

**Pérez-Coria et al. [4]**	2015	Mexico	51(1)	6	Anteverted nostrils/Short Nose; bottom lip fold; brachydactyly/wide fingers; clinodactyly; cryptorchidism; dental malocclusion; dysplastic ears; frontal bossing; hypertelorism; interdigital webbing; joint hypermobility; long philtrum; midface hypoplasia; obesity; shawl scrotum; short stature; simian creases; wide feet; widow's peak
			52(4)	12	Bottom lip fold; brachydactyly/wide fingers; camptodactyly; Cryptorchidism; Dental malocclusion; downward slant palpebral fissures; dysplastic ears; hypertelorism; interdigital webbing; joint hypermobility; Long Philtrum; Midface Hypoplasia; Obesity; Psychomotor retardation; ptosis; shawl scrotum; Short stature; syndactyly; wide feet; widow's peak
			53(5)	4	Anteverted nostrils/Short Nose; bottom lip fold; brachydactyly/wide fingers; camptodactyly; clinodactyly; cryptorchidism; downward slant palpebral fissures; dysplastic ears; hypertelorism; inguiNA†l hernia; interdigital webbing; joint hypermobility; long philtrum; midface hypoplasia; ptosis; shawl scrotum; short stature; simian crease; umbilical hernia; wide feet; widow's peak

**GE et al. [20]**	2015	ChiNA†	54	2	Blepharophimosis; Bone age retarded;Brachydactyly/wide fingers; enlarged liver; groove below upper lip; hypertelorism; inguiNA†l hernia; low set ears; short stature; splenic cyst; swollen testicles
**Parıltay et al. [34]**	2016	Turkey	55	14	Anteverted nostrils/Short Nose;Brachydactyly/wide fingers; broad NA†sal bridge; cryptorchidism; dysplastic ears; hypertelorism; large fontanelle/sutures; ogival palate; shawl scrotum; simian crease; widow's peak
**Griffin et al. [35]**	2016	USA§	56(IV-1)	1	Ankyloglossia;Anteverted nostrils/Short Nose; astigmatism; brachydactyly/wide fingers; clinodactyly; clubbed feet; cryptorchidism; diastema; downward slant palpebral fissures; dysplastic ears; interphalangeal joint contracture; low set ears; metatarsus varus; ptosis; round face; shawl scrotum; simian creases; still's murmur

**Hamzeh et al. [36]**	2017	UAE‡	57(IV-3)	7	ADHD;Anteverted nostrils/Short Nose; blepharophimosis; brachydactyly/wide fingers; broad NA†sal bridge; clinodactyly; Development delayed; downward slant palpebral fissures; dysplastic ears; epicanthal fold; frontal bossing; hypertelorism; interdigital webbing; Low set ears; micrognA†thia; ogival palate; pectus cariNA†tum; pectus excavatum; ptosis; short columella; short neck; short philtrum; Short stature; simian creases; sleep disorder; social/language skills delayed; syndactyly; thin upper lip vermillion; widely spaced nipples; widow's peak
			58(IV-5)	3	ADHD; Anteverted nostrils/Short Nose;Anteverted nostrils/Short Nose; Blepharophimosis; brachydactyly/wide fingers; Broad NA†sal bridge; clinodactyly; Development delayed; downward slant palpebral fissures; dysplastic ears; epicanthal fold; Frontal bossing; hypertelorism; long uvula; Neurocognitive development delayed; overactive frontalis muscle; poor bell's phenomenon; ptosis; short philtrum; short neck; short stature; Short/Broad Hands; telecanthus; widely spaced nipples; widow's peak

Legend

*NR: Not reported; †NA: Not applicable; ‡UAE: United Arab Emirates; §USA: Unite

List of all informations collected from each patient included

Type of mutation	Splicing Mutation	Amino Acid Change	Protein
Missense/Nonsense (1565G>A)	NA†	Arg522His	p.R522H
Missense/Nonsense (1565G>A)	NA†	Arg522His	p.R522H
Gross Deletion (Incl.ex.9-12)	NA†	NA†	NA†

Missense/Nonsense (1829G>A)	NA†	Arg610Gln	p.R610Q
Missense/Nonsense (1829G>A)	NA†	Arg610Gln	p.R610Q
Missense/Nonsense (1829G>A)	NA†	Arg610Gln	p.R610Q

Small Insertion (528insC)	NA†	NA†	NA†
Small Insertion (528insC)	NA†	NA†	NA†
Missense/Nonsense (614G>T)	NA†	Ser205Ile	p.S205I

Small Deletion (982delC)	NA†	NA†	p.(His328Thrfs*32)
Gross Deletions (944-975del32)	NA†	NA†	NA†
Missense/Nonsense (1193 A>C)	NA†	Glu380Ala	p.E380A
Missense/Nonsense (1328 G>A)	NA†	Arg443His	p.R443H

Small Deletion (1316-1319del AGCT)	NA†	NA†	NA†
Small Deletion (2530delG)	NA†	NA†	p.(Val844Trpfs*19)
Missense/Nonsense (c.1223G>A)	NA†	Arg408Gln	p.R408Q

Small Deletion (c.2192delA)	NA†	NA†	p.(Lys731Argfs*132)
Small Deletion (c.2192delA)	NA†	NA†	p.(Lys731Argfs*132)
Small Deletion (c.2192delA)	NA†	NA†	p.(Lys731Argfs*132)
Missense/Nonsense (c.1327G>T)	NA†	Arg443Leu	p.R443L
Missense/Nonsense (c.2221G>T)	NA†	Glu741Term	p.E741*

Missense/Nonsense (c.1396A>G)	NA†	Met466Val	p.M466V
Small Insertions (c.944dupC)	NA†	NA†	p.(Ala316Cysfs*4)
Small Insertions (c.944dupC)	NA†	NA†	p.(Ala316Cysfs*4)

Gross Deletions (ENTIRE GENE)	NA†	NR*	NR*
Small Deletions (806delC)	NA†	NA†	p.(Ala269Valfs*91)
Missense/Nonsense (c.1205G>A)	NA†	Arg402Gln	p.R402Q
Missense/Nonsense (c.1590T>A)	NA†	Tyr530Term	p.Y530*

Small Deletions (1620delC)	NA†	NA†	Asp540Glufs*11
Missense/Nonsense (c.1673C>G)	NA†	Ser558Trp	p.S558W
Splicing (c.1935+3A>C)	IVS11 ds A-C +3	NA†	NA†
Missense/Nonsense (c.1966C>T)	NA†	Arg656Term	p.R656*

Missense/Nonsense (c.1966C>T)	NA†	Arg656Term	p.R656*
Missense/Nonsense (c.1966C>T)	NA†	Arg656Term	p.R656*
Small Deletions (2020_2020 del GAG)	NA†	NA†	Glu676del
Missense/Nonsense (c.2242A>G)	NA†	Lys748Glu	p.K748E

Missense/Nonsense (c.175C>T)	NA†	Gln59Term	p.Q59*
Missense/Nonsense (c.175C>T)	NA†	Gln59Term	p.Q59*
Duplication	NA†	NA†	NA†
Small deletions (c.2016-35delA)	NA†	NA†	NA†

Small deletions (c.2016-35delA)	NA†	NA†	NA†
Small deletions (c.2016-35delA)	NA†	NA†	NA†
Splicing (c.482-2A>G)	IVS2 as A-G -2	NA†	NA†
Missense/Nonsense (c.1468C>T)	NA†	Gln490Term	p.Q490*
Missense/Nonsense (c.1341G>A)	NA†	Trp447Term	p.W447*

Missense/Nonsense (c.1341G>A)	NA†	Trp447Term	p.W447*
Missense/Nonsense (c.1341G>A)	NA†	Trp447Term	p.W447*
Missense/Nonsense (c.1341G>A)	NA†	Trp447Term	p.W447*
Missense/Nonsense (c.1341G>A)	NA†	Trp447Term	p.W447*
Missense/Nonsense (c.1340G>A)	NA†	Trp447Term	p.W447*

Missense/Nonsense (c.1138G>T)	NA†	Glu380Term	p.E380*
Missense/Nonsense (c.1990C>T)	NA†	Gln664Term	p.Gln664*
Missense/Nonsense (c.1990C>T)	NA†	Gln664Term	p.Gln664*

Missense/Nonsense (c.1270A>G)	NA†	Asn424Asp	p.N424D
Splicing Mutations (c.1340+2T>A)	IVS6 ds T-A +2	NA†	NA†
Missense/Nonsense (c.2761C>T)	NA†	Gly757Arg	p.G757R

Small Deletions (c.53delC)	NA†	NA†	p.(Pro18Argfs*106)
Small Deletions (c.53delC)	NA†	NA†	p.(Pro18Argfs*106)

ed States of America; ||bro: Brother; ¶nep= Nephew

tematic review
lfredo Orrico

Insertions/Duplications
NA†
NA†
NA†

NA†
NA†
NA†

NA†
NA†
NA†

NA†
NA†
NA†
NA†

NA†
NA†
NA†

NA†
NA†
NA†
NA†
NA†

NA†
NA†
NA†

NA†
NA†
NA†
NA†

NA†
NA†
NA†
NA†

NA†
NA†
NA†
NA†

NA†
NA†
c.307+5711_2016-227
NA†

NA†
NA†
NA†
NA†
NA†

NA†
NA†
NA†
NA†
NA†

NA†
NA†
NA†

NA†
NA†
NA†

NA†
NA†
